# Solid acid-catalyzed one-step synthesis of oleacein from oleuropein

**DOI:** 10.1038/s41598-023-35423-x

**Published:** 2023-05-22

**Authors:** Yasuhiro Shimamoto, Tadahiro Fujitani, Eriko Uchiage, Hiroko Isoda, Ken-ichi Tominaga

**Affiliations:** 1 National Institute of Advanced Industrial Science and Technology (AIST), Interdisciplinary Research Center of Catalytic Chemistry, Central 5, 1-1-1 Higashi, Tsukuba, Ibaraki 305-8565 Japan; 2 National Institute of Advanced Industrial Science and Technology (AIST), Open Innovation Laboratory for Food and Medicinal Resource Engineering, 1-1-1 Tennodai, Tsukuba, 305-8577 Japan; 3 School of Life and Environmental Science, University of Tsukuba, 1-1-1 Tennodai, Tsukuba, 305-8572 Japan

**Keywords:** Natural product synthesis, Chemistry, Catalysis, Heterogeneous catalysis

## Abstract

In this study, we developed a new synthetic strategy to convert secoiridoid glucosides into unique dialdehydic compounds using solid acid catalysts. Specifically, we succeeded in the direct synthesis of oleacein, a rare component of extra-virgin olive oil, from oleuropein, which is abundant in olive leaves. Whereas the conventional total synthesis of oleacein from lyxose requires more than 10 steps, these solid acid catalysts enabled the one-step synthesis of oleacein from oleuropein. A key step in this synthesis was the selective hydrolysis of methyl ester. Density functional theory calculations at the B3LYP/631+G (d) level of theory revealed the formation of a tetrahedral intermediate bonded to one H_2_O molecule. These solid acid catalysts were easily recovered and reused at least five times by simple cleaning. Importantly, this synthetic procedure was not only applicable to other secoiridoid glucosides, but could also be employed for the corresponding scale-up reaction using oleuropein extracted from olive leaves as the starting material.

## Introduction

**Figure 1:**
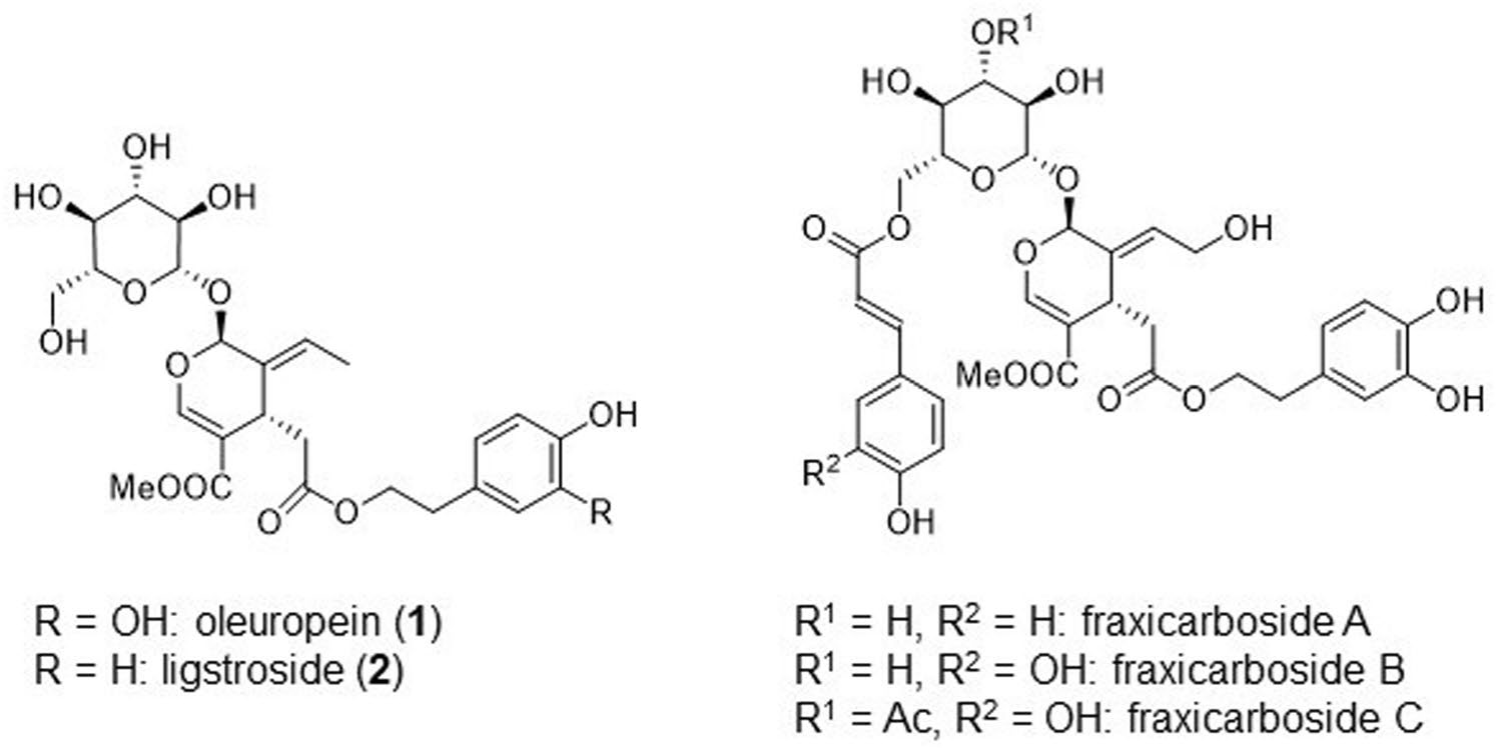
Structures of natural secoiridoids.

The secoiridoid structure is found in many natural products such as oleuropein (**1**)^1, 2^, ligstroside (**2**)^2^, and fraxicarbosides^2^ and is composed of monoterpenoid glycosides with a 2-alkoxydihydropyran skeleton. These monoterpenoid glycosides exhibit antioxidant^3, 4^, antimicrobial^5^, and antitumor activities (Fig. 1)^6^. Moreover, related iridoid glucosides have been often studied in various research areas such as organic synthesis, bioactivity evaluation, compound isolation, and structure determination^7–9^.

Although these secoiridoids are biosynthesized via dialdehydic intermediates in the metabolic pathway^10^, there are very few reports on the bioconversion of secoiridoid glucosides via such dialdehydic intermediates. Oleocanthal (**3**), which is found in extra-virgin olive oil^11^, is a potential natural dialdehyde that exhibits anti-inflammatory and antioxidant activities^11^, reduces β-amyloid accumulation^12^, and inhibits cancer cell growth^13^.

**Figure 2:**
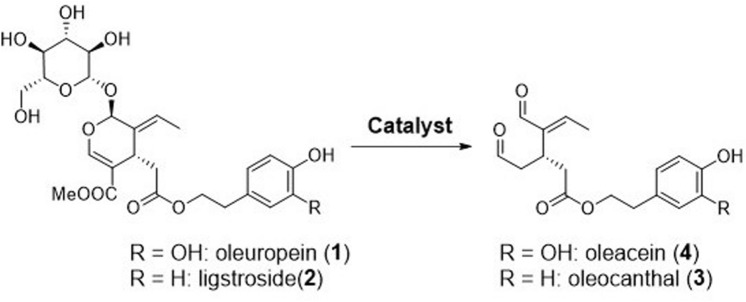
One-step synthesis of oleacein from oleuropein and oleocanthal from ligstroside.

Oleuropein is a common secoiridoid glucoside that is abundant in olive leaves^14, 15^. Herein, we report the direct conversion of oleuropein to oleacein (**4**), a rare component of extra-virgin olive oil, using chemical catalysts (Fig. 2)^16^. This reaction is also applicable to the synthesis of an analogous compound, oleocanthal (**3**), from ligstroside (**2**). Because of the rare occurrence of oleacein, its biological functions have been studied to a lesser extent than those of oleocanthal. Nonetheless, oleacein has been reported to exhibit antioxidant^17–19^ and anti-inflammatory activities^20^, inhibitory activity against angiotensin-converting enzymes related to high blood pressure^21^, protective effects on the damages/metabolic alterations caused by a high-fat diet^22^, and anti-tumor activity in multiple myeloma^23^. Additionally, it can increase the ATP level in a cellular model of early Alzheimer's disease^24^.

Organic synthesis is a promising tool for the synthesis of oleacein because it is difficult to extract large amounts of this compound from natural matrices. Smith et al. reported the total synthesis of oleacein from d-lyxose over 10 steps, with a total yield of 13%^25^. Compared to total synthesis, semi-synthesis is a more efficient, more economical, and greener alternative^26, 27^. Vougogiannopoulou et al. reported the direct conversion of oleuropein into oleacein via Krapcho decarboxylation using two equivalents of sodium chloride; however, the oleacein yield was only 20%^28^. The yield increased to 48% upon microwave heating^29^. Narde et al.^30^ reported the efficient synthesis of natural demethylated oleuropein using Er(OTf)_3_ as a catalyst; demethylated oleuropein was decarboxylated to form oleacein in this synthesis. Therefore, the development of an efficient process to synthesize oleacein from oleuropein can uncover potential implicit functions of oleacein. Moreover, because oleuropein is abundantly present in olive leaves, this strategy will ensure the efficient use of olive wastes.

## Results and discussion

Oleuropein is a known secoiridoid with a 2-alkoxydihydropyran structure, as previously mentioned^1^. Acid hydrolysis of 2-alkoxydihydropyran compounds produces glutaraldehydes under relatively mild conditions such as room temperature^31^. At first, we confirmed the feasibility of the oleuropein-to-oleacein transformation in the presence of homogenous acids.

As a preliminary experiment, we performed the reaction of 13.8 μmol of oleuropein in the presence of 10 mol% hydrochloric acid at 150 °C for 15 h in 0.5 mL of DMSO-*d*_6_ in an NMR tube without stirring. The amount of H_2_O was determined by Karl Fischer titration^32^, and the concentration was adjusted to 75.9 μmol (5.5 equiv of oleuropein). However, neither oleacein nor oleuropein was detected in the reaction mixture after 12 h (Table 1). Interestingly, when the concentration of hydrochloric acid was reduced to 1 mol%, NMR peaks corresponding to oleacein were observed in the spectra, and the yield was 54% based on oleuropein. Further reduction in the concentration of hydrochloric acid to 0.1 mol% increased the yield of oleacein to 67%.

**Table 1:** Conversion of oleuropein to oleacein in the presence of acids.

Acid	Amount (mol%)	Yield of **3**^a^ (%)
HCl	10	None
	1.0	54
	0.1	67
PTSA^b^	10	54
	1.0	73
	0.1	77

Conditions: oleuropein (13.8 μmol), acid, H_2_O (75.9 μmol), DMSO-*d*_6_ (0.5 mL), N_2_ atmosphere, 150 °C, 15 h.

^a^Yield was determined by NMR spectroscopy using tetramethyl benzene as an internal standard.

^b^PTSA, *p*-toluenesulfonic acid.

Thus, the acid hydrolysis of oleuropein not only cleaved the 2-alkoxydihydropyran structure to give a glutaraldehyde structure, but also allowed the simultaneous hydrolysis of methyl ester and decarboxylation to give oleacein. Notably, only a small amount of acid was required for this reaction. To determine the effect of the acid type, we conducted this reaction in the presence of *p*-toluenesulfonic acid (PTSA). Indeed, the serial dilution of PTSA resulted in increased product yield, similar to that observed for hydrochloric acid.

**Figure 3:**
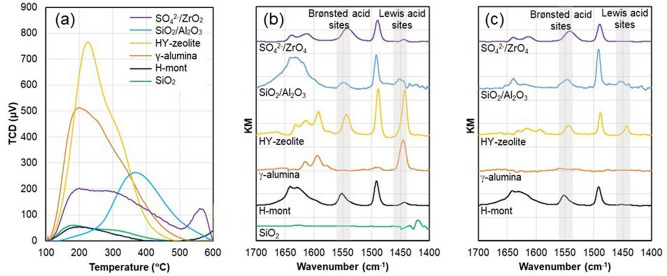
Properties of solid acids. (**a**) Ammonia temperature-programmed desorption profiles. Diffuse reflectance infrared Fourier transform spectra of pyridine absorbed on solid acids after treatment at (**b**) 150 and (**c**) 220 °C.

Based on the above results, we replaced these homogeneous acids with solid acids because the latter is easier to handle and allows better recovery and recycling of the catalysts. Several solid acids with weak and strong acidity were examined; these included proton-exchanged montmorillonite (H-mont), sulfated zirconia (SO_4_^2–^/ZrO_2_), γ-alumina (γ-Al_2_O_3_), proton-exchanged Y-zeolite (HY-zeolite, Si/Al = 5.5), silica-alumina (SiO_2_/Al_2_O_3_), Amberlyst® 70, and silica gel (SiO_2_). Among them, H-mont was prepared from montmorillonite according to a previously reported study, except that the concentration of HCl was changed from 1.1 to 0.22 wt%^33^. The other catalysts were used without any modification. To characterize the solid acid catalysts, Brunauer–Emmett–Teller (BET) analysis and ammonia temperature-programmed desorption (NH_3_-TPD) were conducted for each catalyst. The results are summarized in Table 2, and the NH_3_-TPD profiles are shown in Fig. 3a. The number of acid sites increased in the order H-mont < SiO_2_ < SiO_2_/Al_2_O_3_ < SO_4_^2−^/ZrO_2_ < γ-Al_2_O_3_ < HY-zeolite < Amberlyst 70, the acid site density increased in the order SiO_2_ < SiO_2_/Al_2_O_3_ < HY-Zeolite < H-mont < γ-Al_2_O_3_ < SO_4_^2−^/ZrO_2_ < Amberlyst 70, and the acid strength increased in the order SiO_2_ < γ-Al_2_O_3_ ~ H-mont < HY-Zeolite < SiO_2_/Al_2_O_3_ < SO_4_^2−^/ZrO_2_.

**Table 2:** Characterization of solid acids.

Catalyst	BET surface area (m^2^ g^−1^)	Pore volume (cm^3^ g^−1^)	Total acidity (mmol g^−1^)	Acid site density (× 10^–4^ mmol m^−2^)	NH_3_ desorption peak (°C)
H-mont	52.1	0.041	0.023	4.4	201.0
SO_4_^2−^/ZrO_2_	80.9	0.133	0.152/0.023	18.8/2.8	199.9/560.2
γ-Al_2_O_3_	233.3	0.528	0.287	12.3	199.7
HY-Zeolite	820.8	0.066	0.324	3.9	226.3
SiO_2_/Al_2_O_3_	651.6	0.819	0.142	2.2	368.4
SiO_2_	670.7	0.612	0.032	0.48	183.9
Amberlyst 70^a^	36	–	2.55	708	–

^a^Catalog spec.

The properties of the acid sites of these solid acids were analyzed by diffuse reflectance infrared Fourier transform (DRIFT) spectroscopy using pyridine as a probe molecule. After pretreatment at 150 °C under vacuum for 1 h, followed by pyridine adsorption at room temperature, the solid acids were treated at 150 or 220 °C under vacuum for 12 h. The results of the treatments at 150 and 220 °C are shown in Fig. 3b and c, respectively. As shown in Fig. 3b, no absorption peak in the wavenumber range of 1400–1700 cm^−1^ appeared for SiO_2_, while the other solid acids exhibited almost the same spectra as previously reported^33–36^.

The absorption peak at ~ 1450 cm^−1^ can be assigned to the ring vibration of pyridine coordinated to the Lewis acid sites, while the absorption peak at ~ 1550 cm^−1^ can be assigned to the ring vibration of pyridinium ions bonded to the Brønsted acid sites. The results shown in Fig. 3b and c suggest the following facts: (1) The strength of the acid sites on SiO_2_ was so low that pyridine was desorbed at temperatures below 150 °C. (2) The acid sites of γ-Al_2_O_3_ at 150 °C were mostly Lewis acidic in nature. (3) H-mont, HY-zeolite, SiO_2_/Al_2_O_3_, and SO_4_^2−^/ZrO_2_ had both Lewis and Brønsted acidic sites at 150 °C. (4) The peaks in the spectrum of γ-Al_2_O_3_ treated at 150 °C were not observed for γ-Al_2_O_3_ treated at 220 °C, suggesting that γ-Al_2_O_3_ has the weakest acid strength after SiO_2_. (5) For the other catalysts, the peaks derived from the Lewis acid sites were weaker or absent when they were treated at 220 °C, while those derived from the Brønsted acid sites were observed, indicating that the acid strength of the Lewis acid sites is weaker than that of the Brønsted acid sites. (6) For SiO_2_/Al_2_O_3_, the peak derived from the Brønsted acid sites was slightly larger when it was treated at 220 °C, suggesting the appearance of new Brønsted acid sites.

The reactions in the presence of these solid acids were conducted at 150 °C for 15 h in DMSO-*d*_6_ in an NMR tube without stirring. The results are summarized in Table 3. All these solid acids, including SiO_2_—the weakest solid acid examined, catalyzed the conversion of oleuropein into oleacein with yields in the range of 40–82%. The yields increased in the order γ-Al_2_O_3_ < SiO_2_ < Amberlyst 70 < SiO_2_/Al_2_O_3_ < SO_4_^2−^/ZrO_2_ < HY-zeolite < H-mont. These results suggest that the solid acids with relatively weak to moderately strong Brønsted acidic sites and moderate acid density, such as H-mont and HY-zeolite, afford better yields of oleacein.

**Table 3:** Screening of solid acid catalysts.

Solid acid catalyst	Yield of **4**^a^					
	1st run	2nd run^b^	3rd run	4th run	5th run	After calcination^c^
H-mont	82	82	77	85	77	–
SiO_2_	62	–	–	–	–	–
SiO_2_/Al_2_O_3_	70	74	54	12	–	78
γ-Al_2_O_3_	40	–	–	–	–	–
HY-zeolite	78	57	20	–	–	76
SO_4_^2−^/ZrO_2_	71	65	15	–	–	71
Amberlyst 70	67	67	78	80	78	–

Conditions: oleuropein (13.8 μmol), solid acid (20.0 mg), H_2_O (75.9 μmol), DMSO-*d*_6_ (0.5 mL), N_2_ atmosphere, 150 °C, 15 h.

^a^Yield was determined by NMR spectroscopy using tetramethyl benzene as an internal standard.

^b^Recycling reactions were carried out after washing with methanol and acetone, followed by drying at 110 °C for 12 h.

^c^Calcination was carried out at 600 °C for 6 h in air.

In particular, H-mont exhibited the best recyclability and afforded the highest yield for this reaction despite its relatively weak acid strength and lower acid density (Table 3)—the yield decreased only slightly even after the fifth run by simple washing and drying treatment of the catalyst. In the case of stronger solid acids such as SiO_2_/Al_2_O_3_, HY-Zeolite, and SO_4_^2−^/ZrO_2_, although the oleacein yields were relatively high in the first run, they decreased with increasing number of runs. This decrease was attributed to the accumulation of organic compounds on the catalyst surface owing to the stronger acid sites because the catalytic activity could be recovered by calcination of the catalysts at 600 °C.

Although the hydration of Lewis acids is known to impart Brønsted acidity, γ-Al_2_O_3_, which has mostly Lewis acid sites, afforded the lowest oleacein yield. This was attributed to the weak acid strength of γ-Al_2_O_3_ without pretreatment at high temperature, as indicated by the DRIFT spectra. Moreover, γ-Al_2_O_3_ is known to be converted to the less acidic boehmite, accompanied by a structural change, under the hydration conditions^37, 38^.

**Figure 4:**
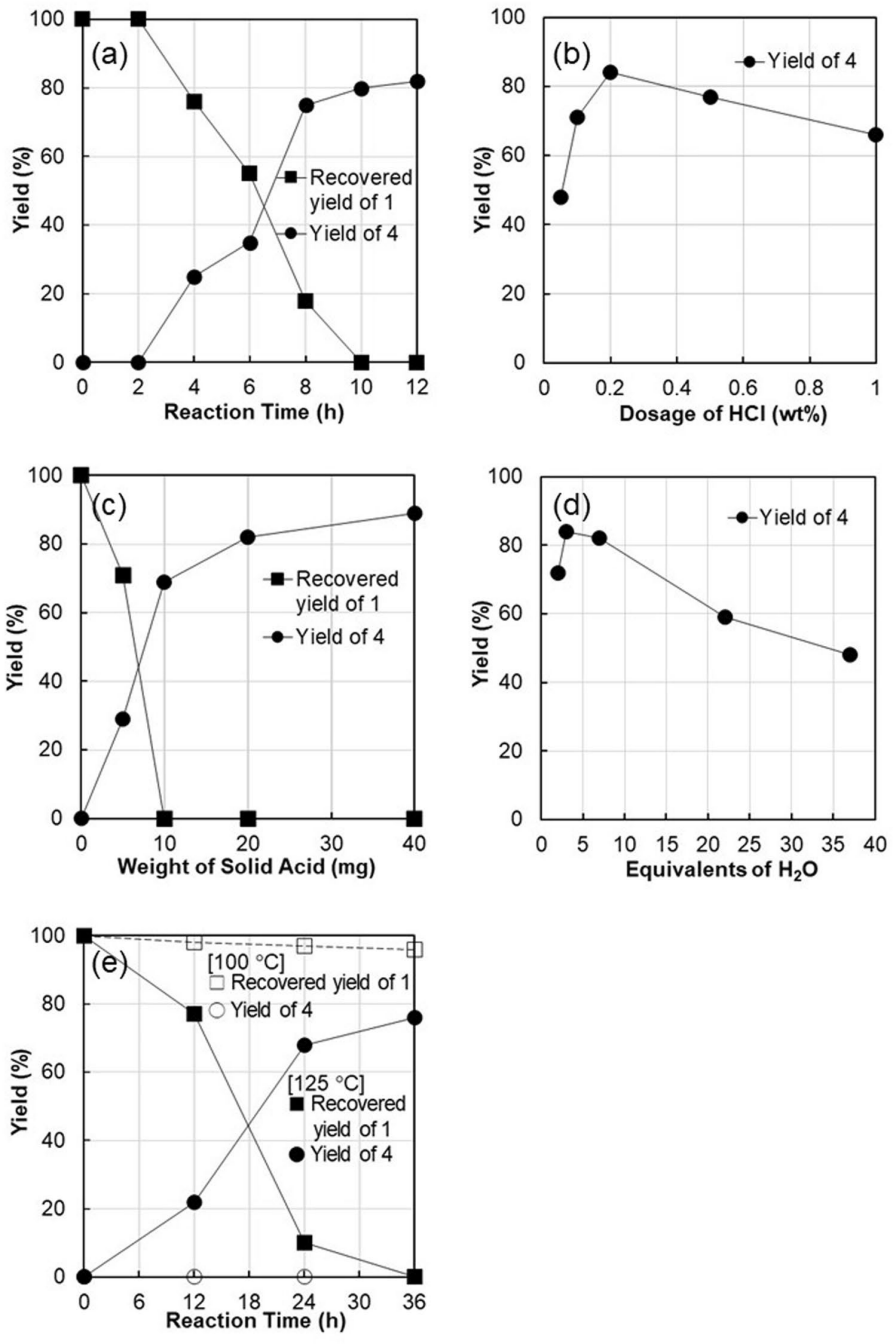
Effect of various factors on the reaction. (**a**) Time course of the H-mont-catalyzed formation of **4** at 150 °C. Effects of (**b**) hydrochloric acid dosage in the preparation of H-mont, (**c**) amount of added H-mont, and (**d**) amount of added H_2_O on the yield of **4**. (**e**) Effect of temperature on the time course of the reaction.

The time course of the reaction in the presence of H-mont is shown in Fig. 4a. After an induction period of 2 h, the oleacein yield began to increase sharply with the consumption of oleuropein. After 8 h of reaction, the consumption of oleuropein and yield of oleacein almost reached saturation.

Since the oleacein yield was affected by the concentration of homogeneous acid (Table 1), we investigated the effect of acid concentration used for the preparation of H-mont on the catalytic activity. Because the acid sites of H-mont were formed by ion exchange with H^+^, the number of acid sites correlated with the acid concentration used during the preparation. Figure 4b shows that the oleacein yield increased with increasing concentration of H^+^ ions used for ion exchange. The optimum H^+^ dosage was 0.22 wt% HCl; the oleacein yield decreased with further increase in H^+^ concentration.

Next, the effect of the amount of H-mont on the yield of oleacein was investigated using H-mont prepared in 0.22 wt% HCl (Fig. 4c). In contrast to the results shown in Fig. 4b, no decrease in oleacein yield was observed, although there was a slight increase when the amount of H-mont was doubled (40 mg). Motokura et al.^33^ reported that when montmorillonite was treated with 1.1 wt% HCl, 98.9% of Na^+^ was exchanged with H^+^. Hence, the results shown in Fig. 4b and c suggest that for oleacein synthesis using H-mont as a catalyst, not only the amount of acid, but also the partial substitution of Na^+^ with H^+^, is responsible for efficient catalysis and catalyst recycling.

The amount of H_2_O was the most important factor in this reaction. Figure 4d shows that the oleacein yield strongly depended on the amount of H_2_O added. The conversion of oleuropein into oleacein involves the hydrolysis of one glycosyl bond and one methyl ester bond and the protection of another ester bond of the hydroxytyrosol group against hydrolysis. Theoretically, 2 equiv of H_2_O is required for this reaction, whereas the optimum amount of H_2_O appears to be 3–6 equiv; further increase in the amount of H_2_O decreased the yield significantly, probably because of overhydrolysis of the ester groups. Such selective hydrolysis is an outcome of the underlying reaction mechanism, as described later.

The effect of reaction temperature is shown in Fig. 4e. The reaction at 125 °C was approximately four times slower than that at 150 °C (Fig. 4a), and no reaction occurred when the temperature was lowered to 100 °C.

We next screened the solvents for this reaction (Table 4). The amount of H_2_O in the solvents was measured by Karl Fischer titration, and the concentration was adjusted to 5.5 equiv of oleuropein. After the reaction, oleacein was separated using column chromatography, and the isolated yield was evaluated. DMSO was determined to be the optimal solvent for this reaction. Moderate yields of oleacein were obtained when γ-butyrolactone (GBL) and diethylene glycol dimethyl ether (diglyme) were used as solvents, while no oleacein was formed in dimethyl formamide (DMF), *N*-methyl-2-pyrrolidone (NMP), and 1-octanol. A kind of aldehyde group can be protected from further reaction in DMSO^39^; thus, the same solvation effects, as those imparted by DMSO, would contribute to oleacein stabilization to afford higher yields. The detailed mechanism is described later.

**Table 4:** Effect of solvent.

Solvent	Yield of **4** (%)^a^
DMSO	80
GBL	56
Diglyme	32
DMF	0
NMP	0
1-octanol	0

Conditions: oleuropein (13.8 μmol), solid acid (20.0 mg), H_2_O (75.9 μmol), solvent (0.5 mL), N_2_ atmosphere, 150 °C, 12 h.

^a^Isolated yield.

**Figure 5:**
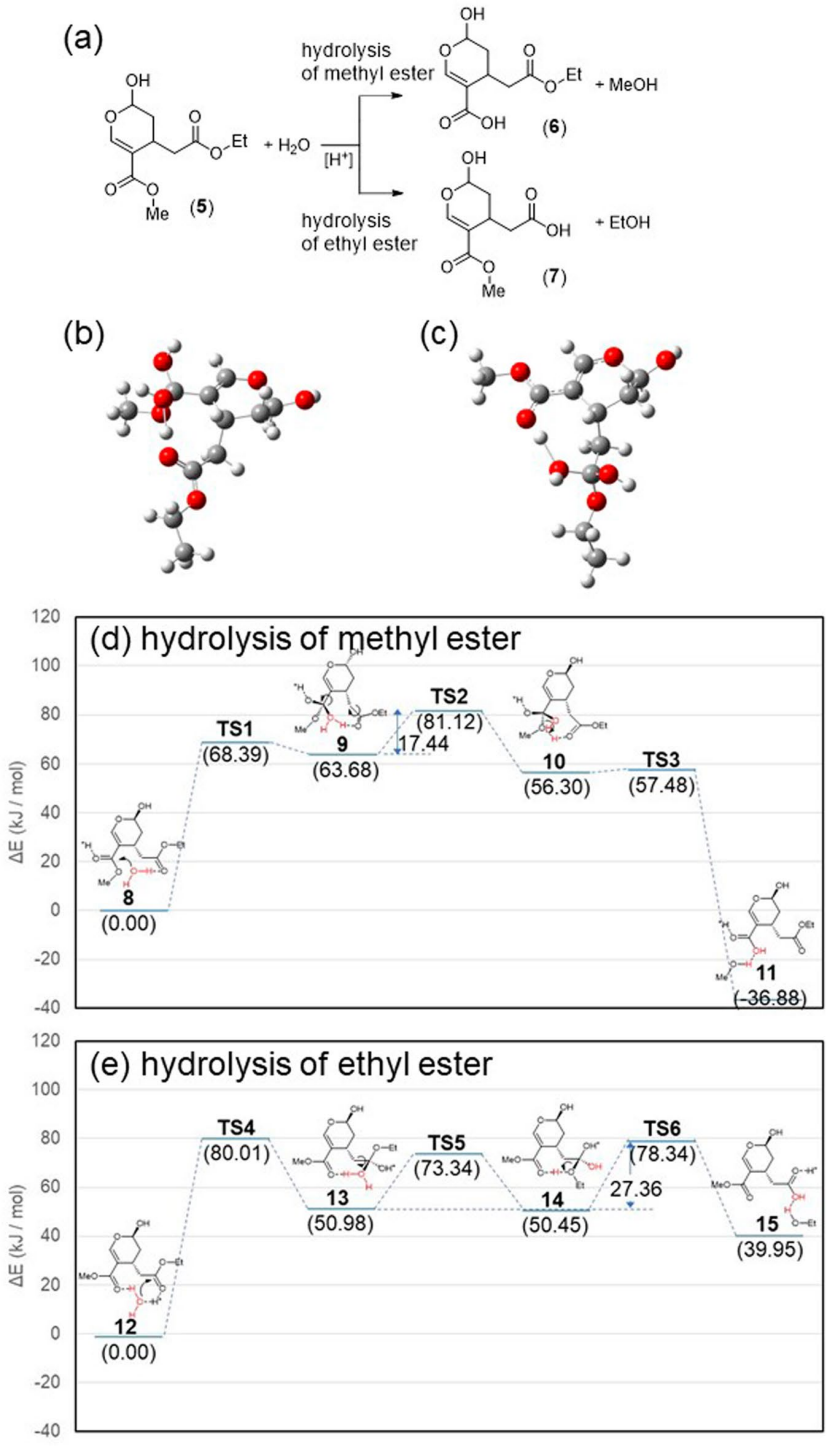
Model reaction of acid hydrolysis of oleuropein and its mechanism. (**a**) Hydrolysis of methyl ester and ethyl ester. Tetrahedral intermediates in the hydrolysis of (**b**) methyl ester and (**c**) ethyl ester. Free-energy profiles of the acid hydrolysis of (**d**) methyl ester and (**e**) ethyl ester.

Oleuropein has two ester groups, methyl ester and hydroxytyrosol ester, which may be involved in the synthesis of oleacein from oleuropein. However, only the methyl group must be hydrolyzed and decarboxylated to give oleacein. To elucidate the reaction mechanism for this selective hydrolysis, we carried out density functional theory (DFT) calculations at the B3LYP/631+G(d) level on the hydrolysis of both groups^40, 41^. To simplify the structure, we considered the acid hydrolysis of model compound **5** (2-hydroxy-3*H*-4-methylene-ethylcarboxylate-5-methylcarboxylatepyrane), as shown in Fig. 5a. In model compound **5**, a hydroxytyrosol group of oleurpein is replaced with the simpler ethyl group since the catechol unit has negligible effect on the acid hydrolysis.

Generally, in the acid hydrolysis of esters, a tetrahedral intermediate is considered to be formed from one carboxylic acid molecule and one H_2_O molecule. Hori et al.^42^ performed theoretical calculations to elucidate the mechanism of this reaction and reported that the inclusion of two H_2_O molecules as reactants is necessary to obtain a tetrahedral intermediate. However, in the acid hydrolysis of the methyl ester group and ethyl ester group of model compound **5**, we found that tetrahedral intermediates **9** and **13** were formed with one H_2_O molecule (Fig. 5b and c). In these tetrahedral intermediates, the H_2_O molecule bonded to each ester group was stabilized by the carbonyl oxygen atom of the other ester group.

The reaction mechanism based on these tetrahedral intermediates and the corresponding free-energy profile are shown in Fig. 5d and e, respectively. The structures of all intermediates and transition states were optimized, and their detailed structures are shown in the Supplementary Information. In the case of methyl ester hydrolysis, rotation of the ethyl ester of **9** enabled proton migration from H_2_O to the methoxy group to form **10**, followed by elimination of methanol to give **11**. On the other hand, in the case of ethyl ester hydrolysis, the rotation of the ethyl ester of **13** enabled proton migration from H_2_O to the ethoxy group to form **14**, followed by elimination of ethanol to give **15**.

The activation energies for methyl ester hydrolysis (81.12 kJ mol^−1^) and ethyl ester hydrolysis (80.01 kJ mol^−1^) were almost same. In both reactions, the rate-determining step was the formation of tetrahedral intermediates **9** and **13**. After the formation of the tetrahedral intermediates, the activation energies were 17.44 and 27.36 kJ mol^−1^ for methyl ester hydrolysis and ethyl ester hydrolysis, respectively. Efficient hydrolysis is enabled by the cooperative effect of the ethyl and methyl esters, which facilitated the migration of protons from H_2_O to the alkoxy groups. Methyl ester hydrolysis is more favorable than ethyl ester hydrolysis because the free energy is lower at the product side for the former, while it is lower at the reactant side for the latter. In the former case, the π-conjugated bond is extended by releasing methanol, which is responsible for the thermodynamic stability at the product side.

**Figure 6:**
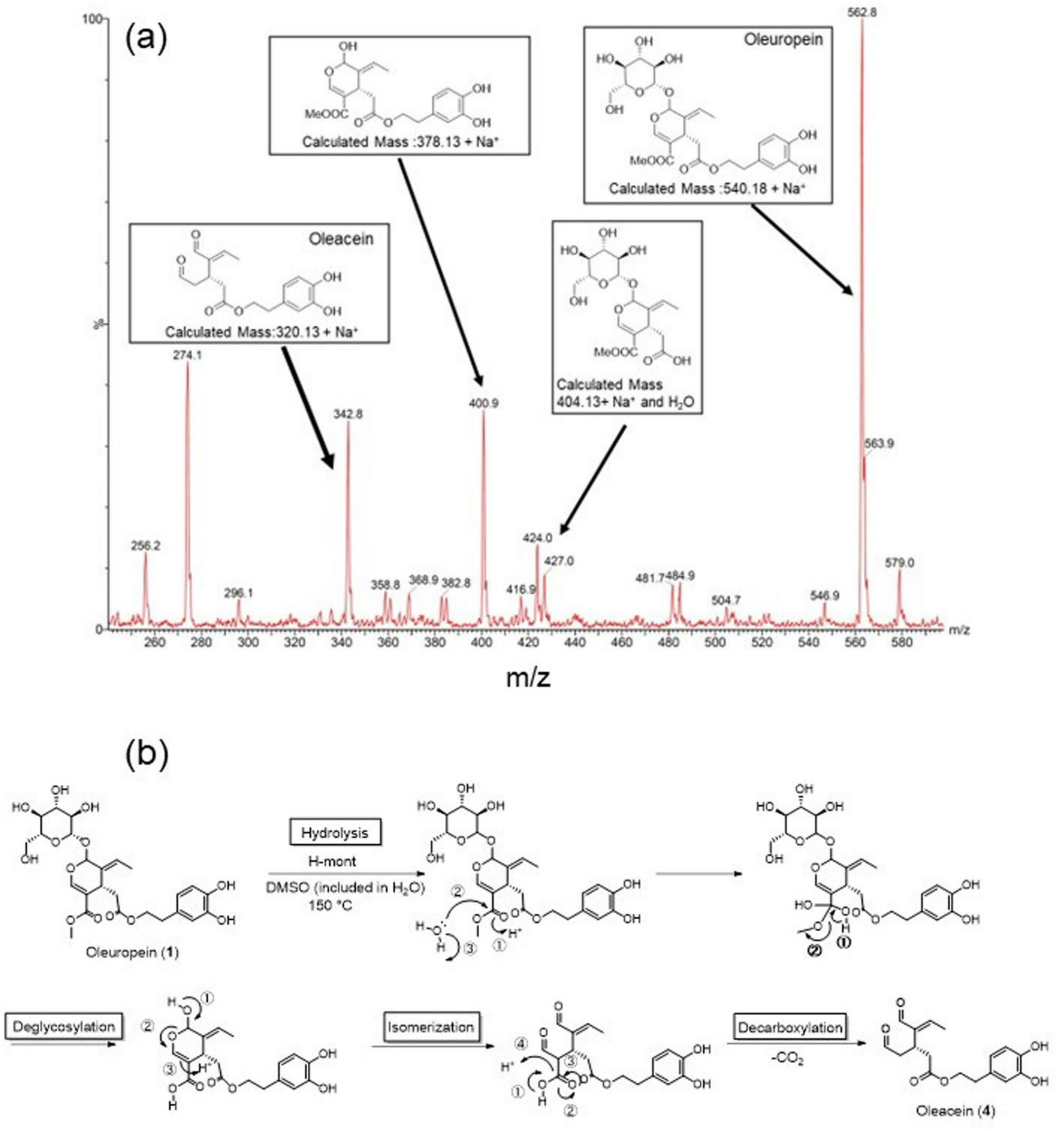
(**a**) ESI-mass spectrum of the reaction solution and (**b**) proposed reaction mechanism.

These theoretical results suggest that the hydrolysis of the methyl ester in oleuropein is more favorable than the hydrolysis of the hydroxytyrosol group. To confirm the intermediates of this reaction, we carried out the reaction at 150 °C for 3 h and examined the reaction solution using ESI–MS in the positive ion mode. Several related compounds (Fig. 6a) were detected in the reaction solution: oleacein (*m*/*z* 342.8, Na^+^ adduct), deglycosylated oleuropein (*m*/*z* 400.9, Na^+^ adduct), a compound in which methyl ester was de-esterified from oleuropein (*m*/*z* 427.0, Na^+^ adduct), and oleuropein (*m*/*z* 562.8, Na^+^ adduct). However, the formation of the demethylesterified form of oleuropein was not observed.

Based on these observations, a reaction mechanism is proposed (Fig. 6b). First, the methyl ester of oleuropein is hydrolyzed to produce carboxylic acid. The activation energy of this step is so low that the hydroxytyrosol group remains unhydrolyzed. After deglycosylation, the 2-hydroxydihydropyran compound is readily isomerized to glutaraldehyde, followed by decarboxylation to give oleacein. A similar transformation of demethylated oleuropein to oleacein proceeds in the presence of a Lewis acid catalyst under mild conditions^30^.

To understand the protective effect of DMSO on the aldehyde group, Tsilomelekis et al. theoretically examined the influence of DMSO solvation on 5-hydroxymethylfurfural (HMF). Their results showed that the solvation of HMF by DMSO increases its LUMO energy, which reduces its susceptibility to nucleophilic attack and minimizes undesirable side reactions^39^. On the other hand, solvation by H_2_O decreases the LUMO energy of HMF, which increases its susceptibility to nucleophilic attack by other molecules.

**Figure 7:**
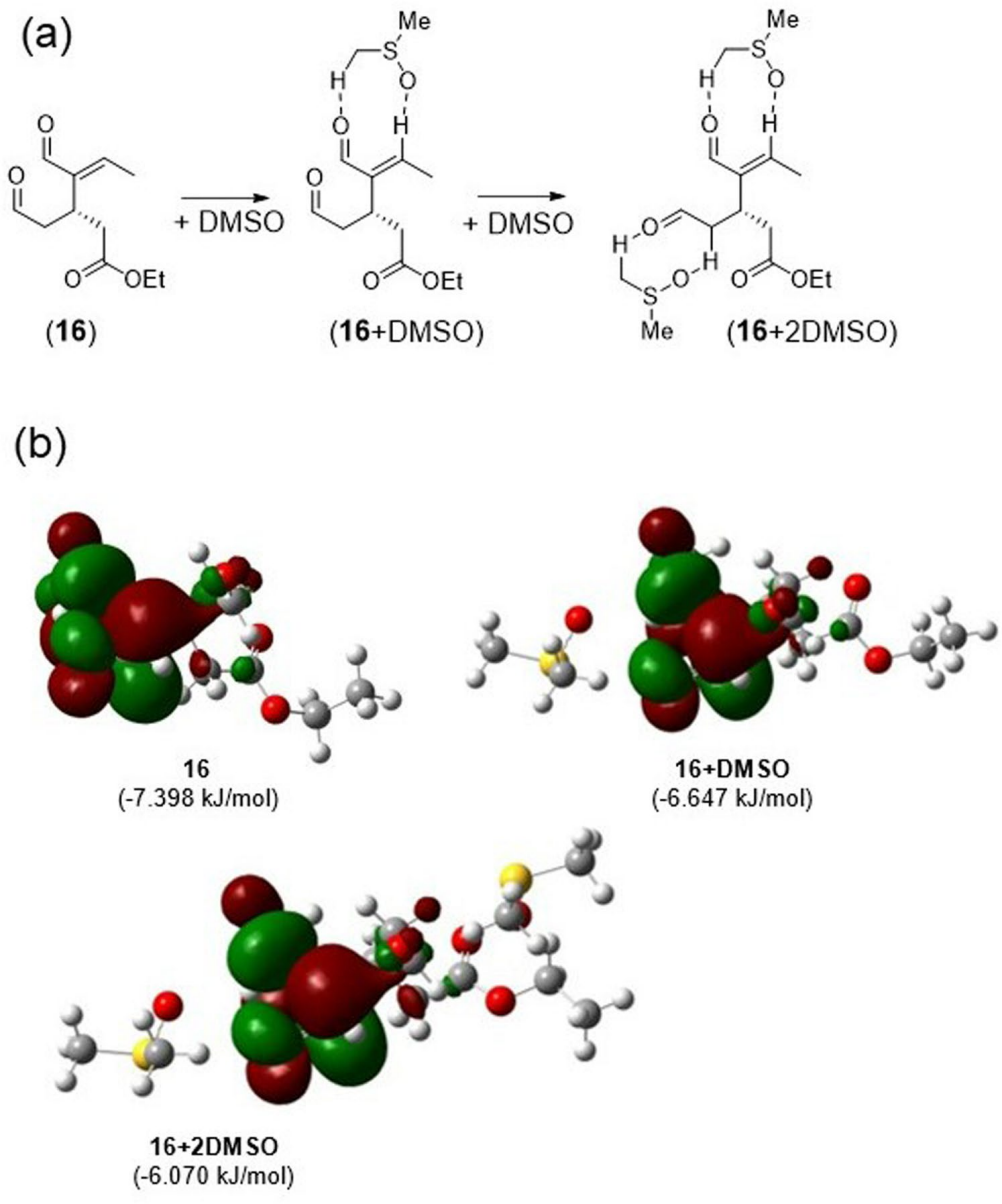
Protective effect of DMSO on a model compound of oleacein. (**a**) Solvation of model compound **16** by DMSO. (**b**) Optimized structures and topologies of the LUMOs of model compound **16** and its adducts with one (**16+DMSO**) and two (**16+2DMSO**) DMSO molecules. The values in parenthesis indicate the LUMO energies.

Thus, we conducted DFT calculations at the B3LYP/631+G(d) level to understand the effect of solvation of model compound **16** by DMSO. In model compound **16**, the hydroxy group of oleacein is replaced with an ethyl group. Because this compound has two aldehyde groups, similar to that in oleacein, two molecules of DMSO can solvate **16**, as shown in Fig. 7a.

Figure 7b shows the optimized structure and topology of the LUMO of model compound **16** solvated by a single DMSO molecule and by two DMSO molecules. As shown for HMF solvation by DMSO, the LUMO topology was not strongly affected by the interaction with DMSO. For all cases, LUMO was the antibonding orbital on the carbonyl and C=C double bond. The main solvation effect imparted by DMSO was the increase in the LUMO energy, which suggests that the solvation of aldehyde groups by DMSO increases the resistance to nucleophilic attack by other molecules. This is considered to be responsible for the higher yield of oleacein in DMSO.

**Figure 8:**
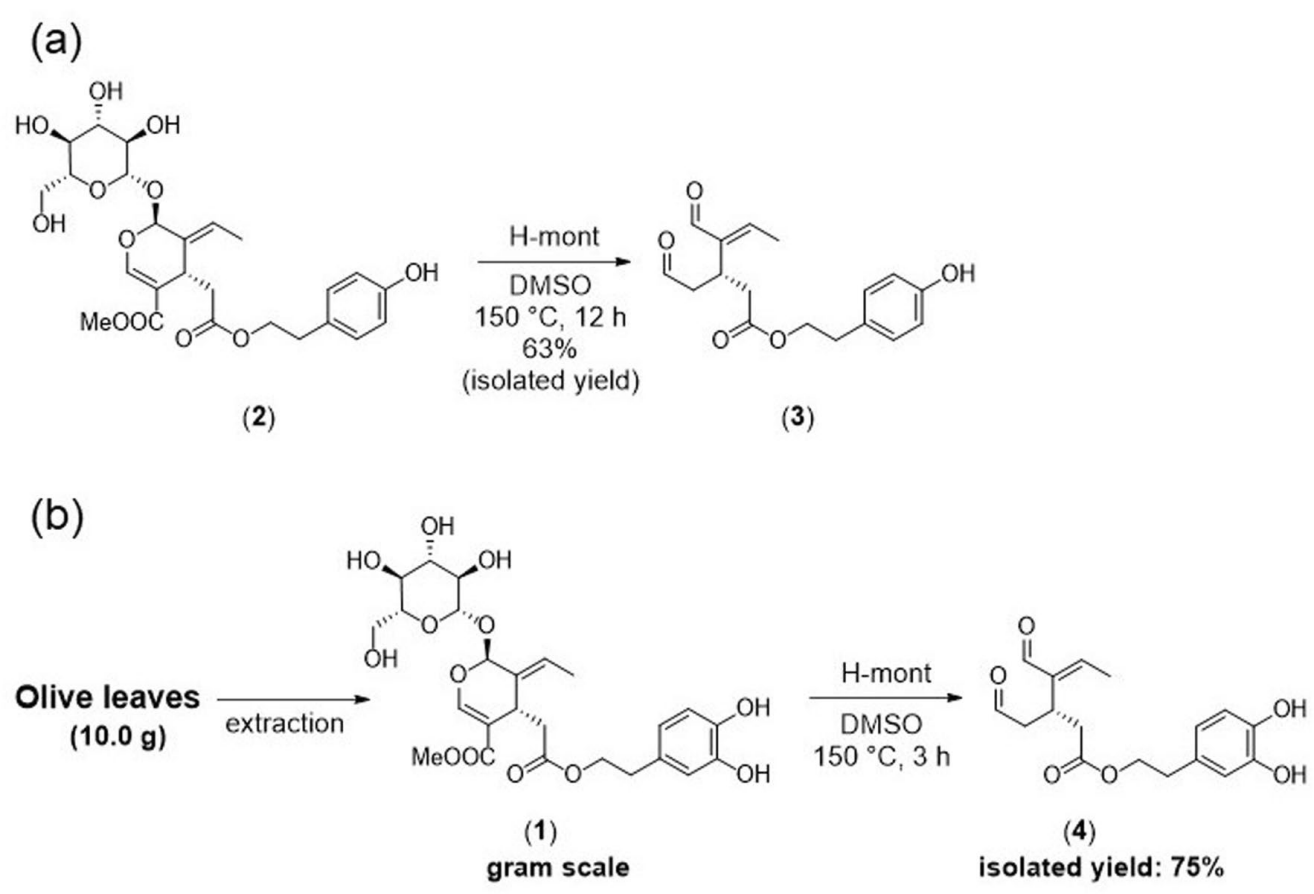
Catalyst applications. (**a**) Synthesis of oleocanthal from ligstroside. (**b**) Scale-up synthesis of oleacein from oleuropein extracted from olive leaves.

To demonstrate an application of this catalysis, we attempted to synthesize oleocanthal from ligstroside using H-mont as a solid acid catalyst. Indeed, oleocanthal was formed in 63% isolated yield when the reaction was conducted at 150 °C for 12 h (Fig. 8a). Compared to the previously reported method using microwave-assisted Krapcho dealkoxycarbonylation, this catalytic reaction can produce oleocanthal in relatively better yield from ligstroside^27^.

We further attempted to scale-up the synthesis of oleacein from oleuropein extracted from olive leaves (Fig. 8b). From 10.0 g of olive leaf powder, 1.53 g of oleuropein powder (purity = 88.0%) could be extracted with 40.0 mL of methanol and water (40:10, v/v), followed by rough separation using silica gel column chromatography (CH_2_Cl_2_/MeOH = 10:1). Using this separated powder, the reaction was carried out at 150 °C in the presence of 2.8 equiv of H_2_O, 10 mL of DMSO, and 3.06 g of H-mont in a 50-mL round-bottom flask without stirring. After 3 h, the reaction was finished with complete consumption of oleuropein confirmed by TLC. Oleacein could be isolated in 75% yield (0.598 g). The reaction in the round-bottom flask was completed in a shorter time than that in the NMR tube. In both cases, the solid catalyst was not suspended but remained at the bottom of the vessel. Therefore, the difference in reaction time is due to the difference in the amount of substrate in contact with the catalyst at the bottom of the vessel per unit time by natural convection. In general, the mass flow rate is proportional to the density, cross-sectional area, and mean velocity. The reaction in the round-bottom flask has a higher substrate concentration and larger diameter than that in the NMR tube, which results in an efficient reaction.

In conclusion, we successfully developed an effective method for the synthesis of oleacein from oleuropein using recyclable solid acid catalysts. H-mont was the most effective solid acid catalyst among those examined and could be reused at least five times after simple cleaning and drying treatment. The methyl ester of oleuropein was selectively hydrolyzed, followed by deglycosylation and isomerization to form a carboxylic-acid-containing glutaraldehyde skeleton that was further decarboxylated to give oleacein. This catalytic reaction could also be employed for other secoiridoids—as demonstrated, oleocanthal was synthesized from ligstroside in reasonable isolated yield. In addition, this reaction could be employed for the gram-scale synthesis of oleacein from olive leaf powder. Since oleacein is a rare component of olive oil, this reaction will provide opportunities for the discovery of new and beneficial medicinal applications.

## Methods

### Materials

All reagents were of research grade and used without further purification, except H-mont^33^ and ligstroside^43^, which were synthesized according to previously reported methods. The concentration of HCl was modified in the preparation of H-mont. Olive leaves (geographical origin in Shodoshima, Japan) were commercially purchased from SHIN-SEI Co., Ltd. All local, national, or international guidelines and legislations were adhered to in this study.

### NMR spectroscopy

^1^H NMR spectra were recorded in DMSO-*d*_6_ and CDCl_3_ on a JEOL LA-400 spectrometer. Chemical shifts were expressed in ppm relative to tetramethylsilane (0 ppm) or CHCl_3_ (7.28 ppm). The coupling constants are given in Hz. ^13^C NMR spectra were recorded on the same spectrometer at 100 MHz using the central resonance of CDCl_3_ (*δ*_C_ 77.0 ppm) as the internal reference, unless otherwise stated.

### Mass spectroscopy

ESI–MS was performed on a Waters ZQ-2000 (ESI) instrument. The needle and cone voltage were + 4.0 kV and 50 V, respectively. The sample solution was introduced directly into the apparatus at a flow rate of 20 µL min^−1^.

### Moisture measurement

The amount of water in the solvents was determined by Karl Fischer titration (Metrohm, 899 coulometer).

### BET surface area analysis

Surface areas and pore volumes of the solid catalysts were determined from the N_2_ adsorption–desorption isotherms (BET method) recorded at − 196 °C using a volumetric unit (Micromeritics ASAP 2020). Prior to adsorption measurements, each catalyst was degassed at 350 °C for 10 h under reduced pressure.

### Ammonia temperature-programmed desorption (NH_3_-TPD)

To investigate the acidity of the solid acids, NH_3_-TPD was conducted on a BELCAT-B chemisorption analyzer (BEL, Japan). The catalyst (0.10 g) was pretreated at 500 °C for 1 h under He flow (50 mL min^−1^). After cooling to 100 °C under He flow, the catalyst was exposed to 5% NH_3_–He (50 mL min^−1^) at 100 °C for 0.5 h. The physisorbed NH_3_ was removed using He flow for 0.25 h at the same temperature. Finally, TPD was performed by heating the catalyst to 610 °C at a rate of 10 °C min^−1^ under He flow (30 mL min^−1^).

### Diffuse reflectance infrared Fourier transform (DRIFT) spectroscopy of adsorbed pyridine

The samples were prepared according to a previously reported method^33^. In a Schlenk flask, the solid acid (100 mg) was pretreated under vacuum at 150 °C for 1 h, and dehydrated pyridine (1.0 mmol) was subsequently introduced under N_2_ atmosphere. The flask was left at room temperature for 3 h to allow the reaction system to reach equilibrium, followed by evacuation of excess pyridine under vacuum at 150 or 220 °C for 12 h. Samples without pyridine treatment were also prepared. DRIFT spectra were recorded on a JASO FT/IR 6800 instrument equipped with a diffuse reflectance unit. The resolution was 1 cm^−1^, and the number of scans was set to 64. Difference spectra were obtained by subtracting the sample spectra without pyridine from those containing pyridine. The Kubelka–Munk function was used to calculate the absorption intensity.

### Synthesis of oleacein from oleuropein

Oleuropein (10 mg, purity > 75%, 0.0138 mmol) was dissolved in DMSO (0.5 mL), which had a water content of 1.36 mg (0.076 mmol). After adding H-mont (20 mg), the reaction tube was filled with N_2_ and left to stand in an oil bath at 150 °C for 12 h without stirring. Following this, the organic layer was washed with water, extracted with AcOEt, dried over Na_2_SO_4_, filtered, and concentrated. The residue was purified using silica gel chromatography (hexane/AcOEt = 10:1 to 1:1) to obtain 3.5 mg of oleacein (isolated yield: 80%) as a yellow powder.

### Computational study

We carried out DFT calculations using the B3LYP/631G+(d) method with the Gaussian 09W program^44^. All optimized species were verified as either minima or transition structures based on the presence of zero or a single imaginary vibrational frequency. Intrinsic reaction coordinates were examined to confirm that the transition state structure connected the correct reactant and product on the energy surface.

## Supplementary Information

Supplementary Information.

## Data Availability

Detailed data for the syntheses of all compounds and catalysts, NMR (^1^H and ^13^C) data of the products, and calculated results for the theoretical calculations of compounds **5–16, TS1**–**TS6**, **16+DMSO**, and **16+2DMSO** can be found in the Supplementary Information.
