# Supplementary material for: Solid acid-catalyzed one-step synthesis of oleacein from oleuropein
Source: Sci Rep. 2023 May 22;13:8275. doi: 10.1038/s41598-023-35423-x (PMC10203140; doi:10.1038/s41598-023-35423-x)
Supplement: Supplementary file 1 — Supplementary Information. [file 41598_2023_35423_MOESM1_ESM.pdf]

Supplementary Information

Solid acid-catalyzed one-step synthesis of oleacein from oleuropein

Yasuhiro Shimamoto[1], Tadahiro Fujitani[1], Eriko Uchiage[2], Hiroko Isoda[2,3], Ken-ichi Tominaga[1,2,]*

[1]National Institute of Advanced Industrial Science and Technology (AIST), Interdisciplinary Research Center of Catalytic Chemistry (IRC3), Central 5, 1-1-1 Higashi, Tsukuba, Ibaraki 305-8569, Japan
[2]National Institute of Advanced Industrial Science and Technology (AIST), Open Innovation Laboratory for Food and Medicinal Resource Engineering (FoodMed-OIL), 1-1-1 Tennodai, Tsukuba 305-8577, Japan
[3]School of Life and Environmental Science, University of Tsukuba, 1-1-1 Tennodai, Tsukuba 305-8572, Japan

E-mail: k-tominaga@aist.go.jp

Table of Contents

1. **General Information**
   - 1.1 Materials
   - 1.2 NMR spectroscopy
   - 1.3 Mass spectroscopy
   - 1.4 Other analyses
2. **Catalyst preparation and synthesis**
   - 2.1 Preparation of proton-exchanged montmorillonite
   - 2.2 Synthesis of oleacein and oleocanthal
     - 2.2.1 Synthetic procedure of oleacain with hydrochloric acid and *p*-toluenesulfonic acid
     - 2.2.2 Synthetic procedure of oleacein with solid acid catalysts
     - 2.2.3 Synthetic procedure of oleacein using isolated oleuropein from olive leaves
     - 2.2.4 Synthesis of oleocanthal with proton-exchanged montmorillonite from ligstroside
     - 2.2.5 HPLC charts
3. **DFT calculation results (8 - 16, TS1 - TS6, 16+DMSO, 16+2DMSO)**

1. General Information

1.1 Materials

All reagents were of research grade and used without further purification. TLC was performed on silica gel (60 F-254, 0.25 mm Plates). Column chromatography was carried out on Silica gel 60N (spherical, neutral, particle size 100-210 μm, Kanto Chemical Co., Inc.)

Oleuropein was purchased from Toronto Research Chemicals. Na-montmorillonite (Kunipia F) was purchased from Kunimine Industry in Japan. Sulfuric acid/Zirconium, Zirconium dioxide, Silica Alumina, γ-Aluminum oxide was purchased from FUJIFILM Wako Pure Chemical industry. Y-exchanged Zeolite (HSZ-320HOA) was purchased from TOSO industry. Amberlyst® was purchased from Organo corporation. Silica gel and dimethyl sulfoxide-d6 were purchased from Sigma-Aldrich. Hydrochloric acid, *p*-toluenesulfonic acid and dimethyl sulfoxide were purchased from Kishida Chemical. Olive leaves was purchased from Hinata Food/SINSEI KOSAN company.

Olive leaves were from Shodoshima, Japan, and were purchased commercial from SHIN-SEI Co. Ltd. All local, national or international guidelines and legislation were adhered to in this study.

1.2 NMR spectroscopy

^{1}H NMR spectra were recorded in DMSO-d6 and $CDCl_3$ on JEOL LA-400 spectrometer. Chemical shifts are expressed in ppm relative to tetramethylsilane (0 ppm) or $CHCl_3$ (7.28 ppm). The coupling constants are given in Hz. ^{13}C NMR spectra were recorded on the same spectrometers at 100 MHz, using the central resonance of $CDCl_3$ (δ_C 77.0 ppm) as the internal reference unless otherwise stated.

1.3 Mass spectroscopy

Low-resolution mass spectra (LRMS) were obtained on a Waters ZQ-2000 (ESI). The needle and cone voltage were +4.0 kV and 50 V, respectively. The sample solution was directly introduced into the apparatus at a flow rate of 20 μL/min.

1.4 Other analyses

Optical rotations were determined with a JASCO DIP-1000. The amount of water in DMSO was measured by Karl Fischer titration (Metrohm, 899 coulometer). The purity of test compounds was determined by analytical HPLC. For analytical HPLC, unless otherwise noted, a Discovery® HS C18 HPLC column (250×4.6 mm I.D, 5 μm, Sigma-Aldrich.) was employed with a linear gradient of water: acetonitrile with gradient from 100:0 to 0:100 in 40 minutes at a flow rate of 1.0 mL/min on a Shimadzu Prominence system (UV, 254 nm).[1,2]

2. Catalyst preparation and synthesis

2.1 Preparation of proton-exchanged montmorillonite

Na^+-montmorillonite was protonated with hydrochloric acid in accordance with the procedure reported by Kaneda et al.[3] A mixture of the Na^+-montmorillonite (9.0 g) and 600 mL of HCl (0.22 wt%) was stirred at 90 °C for 24 hours. The obtained slurry was filtered and washed with deionized water and dried at 110 °C in air, followed by breaking the solid in a mortar to afford proton-exchanged montmorillonite as a gray powder (7.6 g). We also prepared the proton-exchanged montmorillonite catalysts with changing the concentration of hydrochloric acid (1.1, 0.55, 0.11, 0.055 wt%) and used them to investigate the effects of acid amounts in the catalyst.

2.2 Synthesis of oleacein and oleocanthal

2.2.1 Synthetic procedure of oleacain with hydrochloric acid and *p*-toluenesulfonic acid

Oleuropein (10 mg, purity >75%, 0.0138mmol) was dissolved in 0.5 mL of DMSO-d6 in an NMR tube. Its H_2O content was determined to be 1.36 mg (0.076 mmol) by Karl Fischer titration. Subsequently, tetramethyl benzene (0.5 mg, 3.7 μmol) was added to the tube as internal standard, followed by adding hydrochloric acid (10~0.1 mol%, 10 μL) or *p*-toluenesulfonic acid (10~0.1 mol%). This mixture was treated at 150 °C for 12 hours in an oil bath without stirring. After the reaction, NMR spectrum of this mixture was measured to calculate the NMR yield of oleacein relative to oleuropein.

2.2.2 Synthetic procedure of oleacein with solid acid catalysts

Oleuropein (10 mg, purity >75%, 0.0138 mmol) was dissolved in 0.5 mL of DMSO-d6, to which tetramethyl benzene (0.5 mg, 3.7 μmol) was added as an internal standard in an NMR tube. Subsequently, a certain amount of solid acid catalysts was added and the NMR tube was left to stand in oil bath at 150 °C for 12 hours without stirring. After the reaction, NMR spectrum of this mixture was measured to calculate the NMR yield of oleacein relative to oleuropein.

For the catalyst recycle experiments, the used solid catalyst was washed with methanol (5 mL × 3) and acetone (5 mL × 3), followed by drying at 110 °C and was used for the next run without further treatment. If the calcination was required, the catalyst was treated at 600 °C for 6 hours in the electric furnace before using for the next run.

The time course measurement was carried out by removing the reaction tube from oil bath at each time, followed by NMR measurement to calculate the yield of oleacein.

The effects of H_2O were evaluated by the H_2O content in DMSO-d6 being defined. After the remained H_2O in DMSO-d6 was removed with molecular sieve, a certain amount of H_2O was added to adjust the content by checking the H_2O content with Karl Fischer titration.

To investigate the effects of solvents, oleuropein (10 mg, purity >75%, 0.0138 mmol) was dissolved in each solvent in an NMR tube. The H_2O content of each solvent was measured by Karl Fischer

titration and a certain amount of H_2O was added to adjust the H_2O content to the same amount in DMSO (1.36 mg, 0.076 mmol). Subsequently, H-mont (20 mg) was added and the NMR tube was left to stand in oil bath at 150 °C without stirring. After 12 hours, the reaction solution was filtered to remove the catalyst. The organic layer was washed with water and extracted with AcOEt, dried over Na_2SO_4, filtered and concentrated. The residue was purified by silica gel chromatograph (hexane/AcOEt = 10:1 to 1:1) to get oleacein.

2.2.3 Synthetic procedure of oleacein using isolated oleuropein from olive leaves (Eq. S1)

Olive leaves (10 g) were soaked in methanol and water (40 mL, 4:1 v/v) for 12 hours at room temperature in accordance with the previous report.[4] The green exuded solution was filtered and evaporated. The residue was roughly purified by silica gel column chromatography (CH_2Cl_2/MeOH = 10:1) to give (1.53 g, purity 88%, determined at 254 nm wavelength of HPLC) as a green powder.

The powder (1.53 g, containing 2.49 mmol of oleuropein, calculated from 88% purity) was dissolved in DMSO (10.0 mL, containing 7.06 mmol of H_2O, measured by Karl Fischer titration) in a 50 mL round-bottom flask. H-mont (3.06 g) was introduced in the mixture and the flask was left to stand in oil bath at 150 °C. After 3 hours, the complete consumption of oleuropein was confirmed by TLC and the flask was cooled to room temperature. The reaction solution was filtered to remove the catalyst. The organic layer was washed with water and extracted with AcOEt, dried over Na_2SO_4, filtered and concentrated. The residue was purified by silica gel chromatography (hexane/AcOEt = 10:1 to 1:1) to obtain oleacein (598mg, 75%).

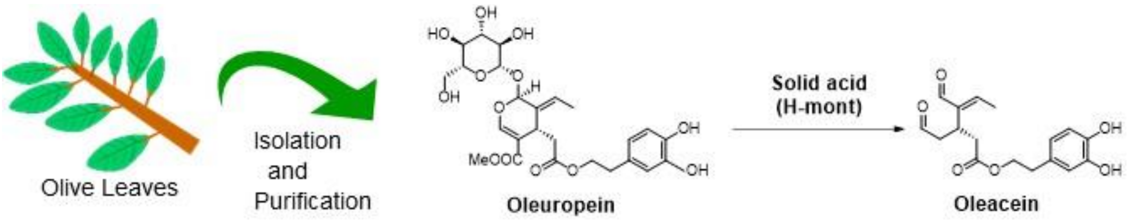

Figure S1. Oleacein synthesis from olive leaves.

(–)-Oleacein; (3*S*,4*E*)-4-Formyl-3-(2-oxoethyl)-4-hexenoic acid 2-(3,4-dihydroxyphenyl) ethyl ester White solid. $[\alpha]^{26}_{D}$ –0.78 (c 0.32, $CHCl_3$); ^{1}H NMR (400 MHz, CDCl3) δ = 9.65 (s,1H), 9.19 (d, *J* = 2.0Hz, 1H), 6.78 (d, *J* = 8Hz, 1H), 6.71 (d, *J* = 2.0 Hz, 1H), 6.65 (q, *J* = 7.2 Hz, 1H), 6.60 (dd, *J* = 2.0, 8.0 Hz, 1H), 4.26-4.12 (m, 2H), 3.67-3.59 (m, 1H), 2.94 (dd, *J* = 18.0, 6.0 Hz, 1H), 2.83-2.80 (m, 3H), 2.78 -2.71 (m, 1H), 2.63 (dd, *J* = 16.0, 6.4 Hz, 1H), 2.06 (d, *J* = 7.6 Hz, 3H); ^{13}C NMR NMR (100MHz, CDCl3) δ = 200.9, 195.7, 171.8, 155.2, 143.3, 143.1, 142.7, 130.4, 121.3, 116.3, 115.2, 65.2, 46.3, 37.0, 34.2, 27.2, 15.3; LRMS (ESI) calced for $C_{17}H_{20}NaO_6$ $[M+Na]^+$: 343.11. Found: 343.1.

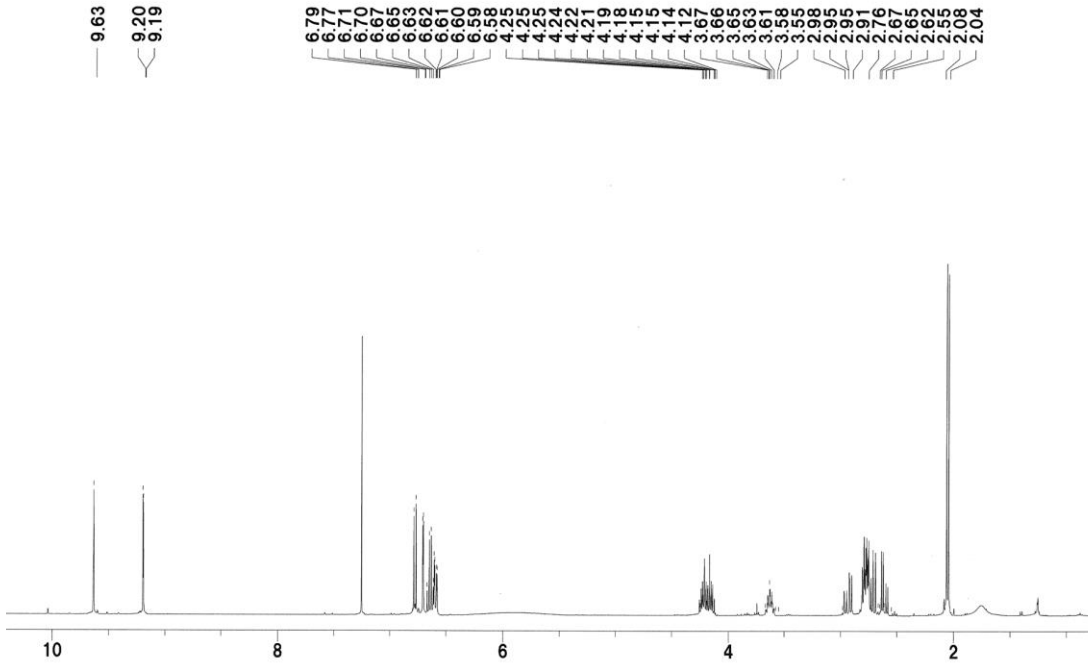

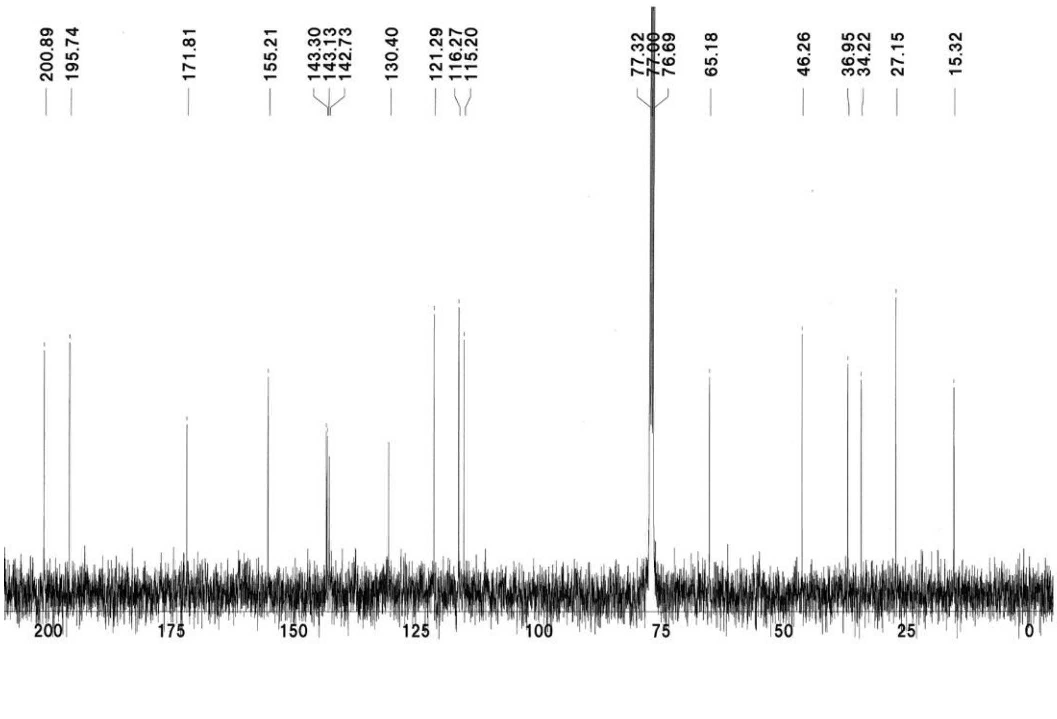

Figure S2. ^{1}H and ^{13}C NMR chart of oleacein.

2.2.4 Synthesis of oleocanthal with proton-exchanged montmorillonite from ligstroside (Eq. S2)

Ligstroside (11 mg, 0.021 mmol) was synthesized according to the reported paper. [5] It was dissolved in 10 mL of DMSO-d6 containing 127 mg (7.06 mmol) of H_2O in an NMR tube. Subsequently, proton-exchanged montmorillonite (22 mg) was added to the tube, which was left to stand in oil bath at 150 °C for 12 hours. After the reaction, the reaction solution was filtered to remove the catalyst. The organic layer was washed with water and extracted with AcOEt, dried over Na_2SO_4, filtered and concentrated. The residue was purified by silica gel chromatography (hexane/AcOEt = 10:1 to 1:1) to afford oleocantal (4.1 mg, 63%).

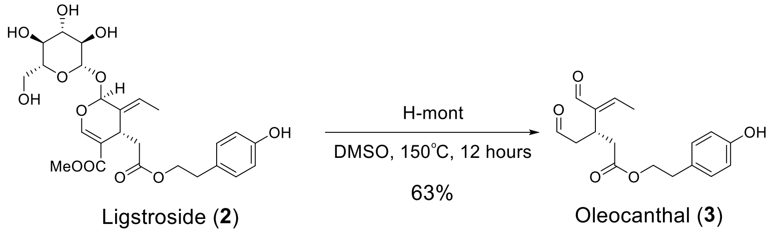

Figure S3. Oleocanthal synthesis from ligstroside.

(–)-Oleocanthal; (3*S*,4*E*)-4-Formyl-3-(2-oxoethyl)-4-hexenoic acid 2-(4-hydroxyphenyl) ethyl ester, Colorless oil. $[\alpha]^{26}_D$ −0.76 (c 0.25, $CHCl_3$) ^{1}H NMR (400MHz, CDCl3) δ 9.64 (s, 1H), 9.24 (d, *J* = 2.0 Hz, 1H), 7.04 (d, *J* = 8.0 Hz, 1H), 6.76 (d, *J* = 8.8 Hz, 1H), 6.64 (q, *J* = 7.1 Hz, 1H), 4.25-4.18 (m, 2H), 3.68-3.58 (m, 1H), 2.99 (m, 1H), 2.82 (t, *J* = 7.0 Hz, 2H), 2.79-2,58 (m, 3H), 2.07 (d, 6.8 Hz, 3H); ^{13}C NMR (100 MHz, CDCl3)δ = 200.4, 195.1, 171.9, 154.3, 154.2, 143.2, 130.0, 129.8, 115.3, 65.1, 46.2, 36.9, 34.2, 27.2, 15.3; LRMS (ESI) calced for $C_{17}H_{18}O_5$ $[M+H]^+$: 305.14. Found: 305.1.

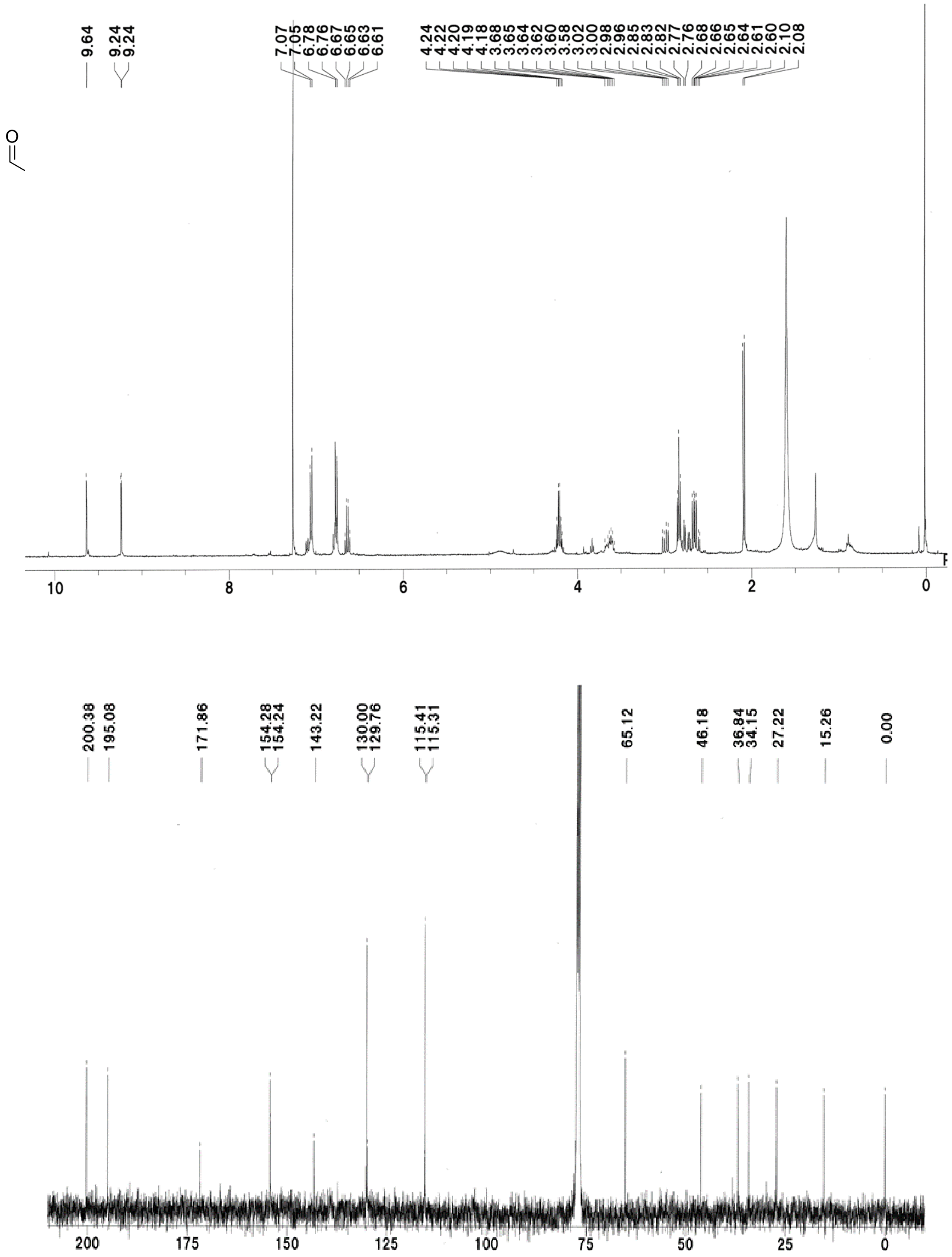

Figure S4. ^{1}H and ^{13}C NMR chart of oleocanthal.

2.2.5 HPLC charts

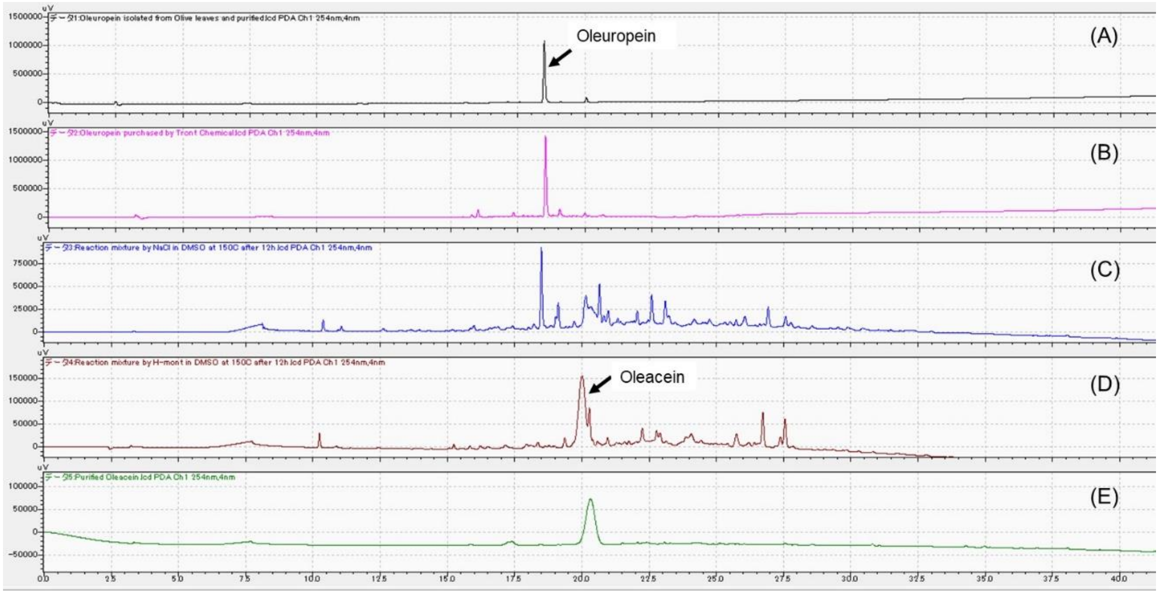

Figure S5. Comparison of HPLC charts. (A) oleuropein (isolated from olive leaves, purity:88%), (B) oleuropein (purchased from Toronto Research Chemicals, purity:75%), (C) reaction solution according to ref. 6 (oleuropein was treated with 2 eq. of NaCl and 10 eq. of H_2O in DMSO at 150 °C for 12 h), (D) reaction solution according to 2.2.3, and (F) purified oleacein from the reaction solution of (D).

3. DFT calculation results (8 - 16, TS1 - TS6, 16+DMSO, 16+2DMSO)

**Compound: 8**		
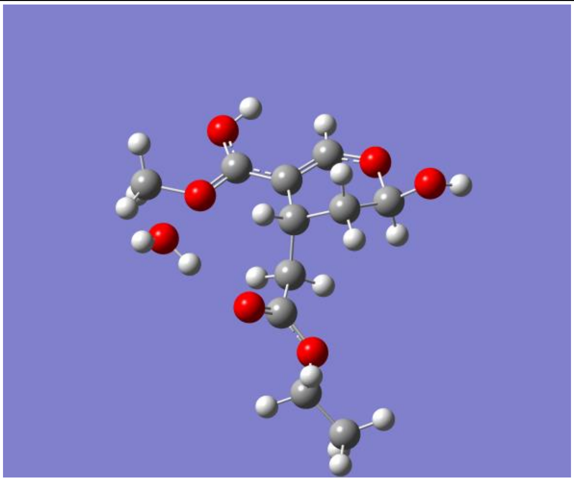	Calculation Type	FOPT
	Calculation Method	RB3LYP
	Basis Set	6-31+G(d)
	Charge	1
	Spin	Singlet
	E(RB3LYP)	-956.96794210
	RMS Gradient Norm	0.00000239
	Imaginary Freq	
	Dipole Moment	6.0173
	Point Group	C1
**Compound: 9**		
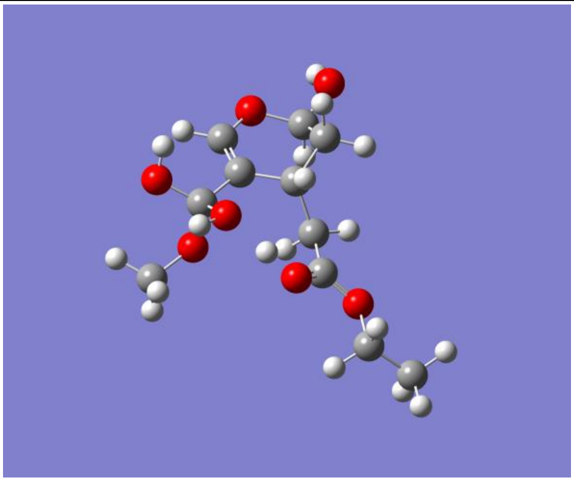	Calculation Type	FOPT
	Calculation Method	RB3LYP
	Basis Set	6-31+G(d)
	Charge	1
	Spin	Singlet
	E(RB3LYP)	-956.94368859
	RMS Gradient Norm	0.00000127
	Imaginary Freq	
	Dipole Moment	7.3566
	Point Group	C1
**Compound: 10**		
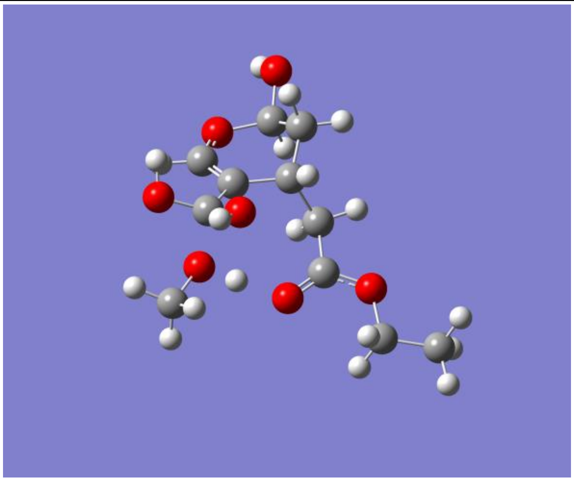	Calculation Type	FOPT
	Calculation Method	RB3LYP
	Basis Set	6-31+G(d)
	Charge	1
	Spin	Singlet
	E(RB3LYP)	-956.94649933
	RMS Gradient Norm	0.00000335
	Imaginary Freq	
	Dipole Moment	3.6209
	Point Group	C1

Compound: 11

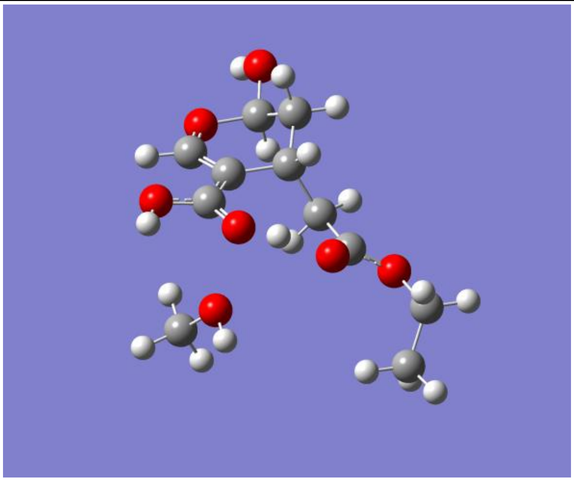

Calculation Type	FOPT
Calculation Method	RB3LYP
Basis Set	6-31+G(d)
Charge	1
Spin	Singlet
E(RB3LYP)	-956.98199
RMS Gradient Norm	1.557e-06
Imaginary Freq	
Dipole Moment	4.1880269
Point Group	C1

Compound: 12

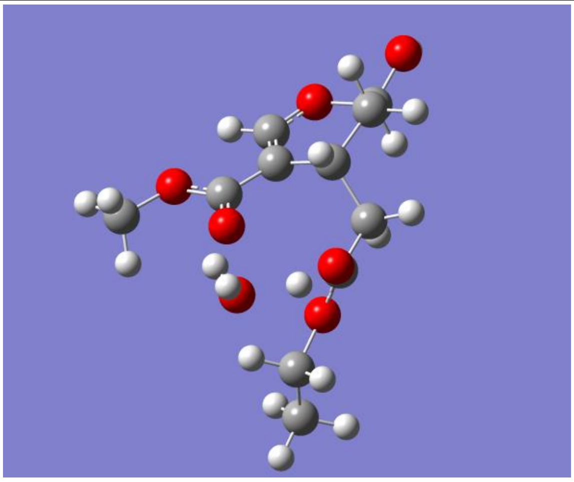

Calculation Type	FOPT
Calculation Method	RB3LYP
Basis Set	6-31+G(d)
Charge	1
Spin	Singlet
E(RB3LYP)	-956.98393
RMS Gradient Norm	2.147e-06
Imaginary Freq	
Dipole Moment	10.918291
Point Group	C1

Compound: 13

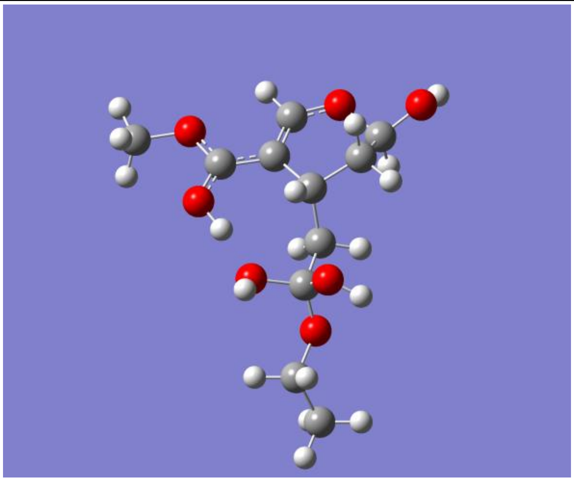

Calculation Type	FOPT
Calculation Method	RB3LYP
Basis Set	6-31+G(d)
Charge	1
Spin	Singlet
E(RB3LYP)	-956.96452
RMS Gradient Norm	3.783e-06
Imaginary Freq	
Dipole Moment	6.2183323
Point Group	C1

**Compound: 14**		
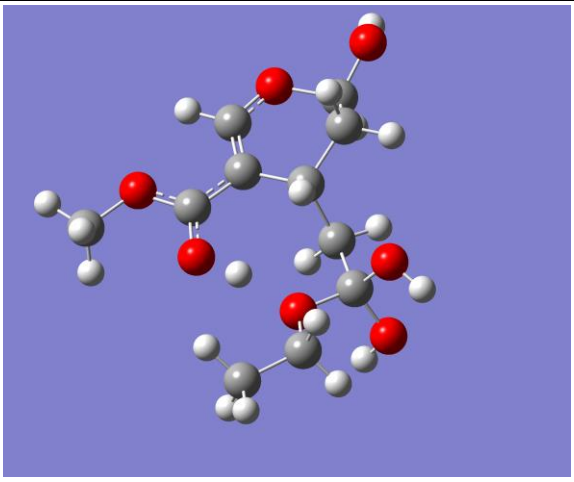	Calculation Type	FOPT
	Calculation Method	RB3LYP
	Basis Set	6-31+G(d)
	Charge	1
	Spin	Singlet
	E(RB3LYP)	-956.96472
	RMS Gradient Norm	6.405e-06
	Imaginary Freq	
	Dipole Moment	13.383306
	Point Group	C1
**Compound: 15**		
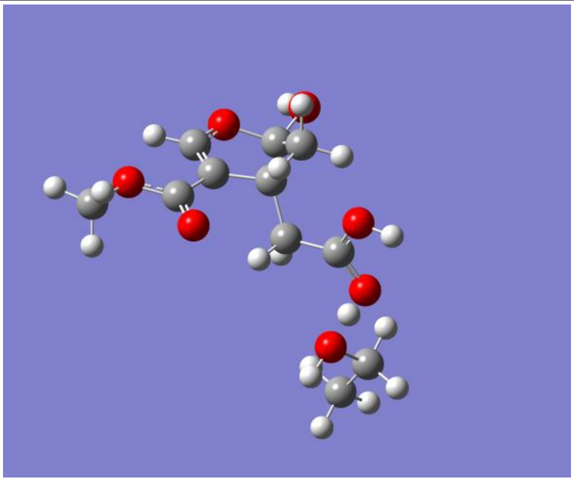	Calculation Type	FOPT
	Calculation Method	RB3LYP
	Basis Set	6-31+G(d)
	Charge	1
	Spin	Singlet
	E(RB3LYP)	-956.96872
	RMS Gradient Norm	4.23e-06
	Imaginary Freq	
	Dipole Moment	14.687611
	Point Group	C1
**Compound: TS-1**		
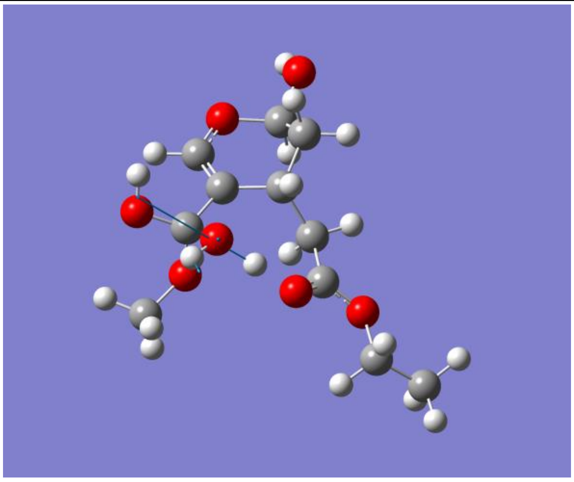	Calculation Type	TS/FREQ
	Calculation Method	RB3LYP
	Basis Set	6-31+G(d)
	Charge	1
	Spin	Singlet
	E(RB3LYP)	-956.94189
	RMS Gradient Norm	3.022e-06
	Imaginary Freq	1
	Dipole Moment	4.3081686
	Point Group	C1

**Compound: TS-2**		
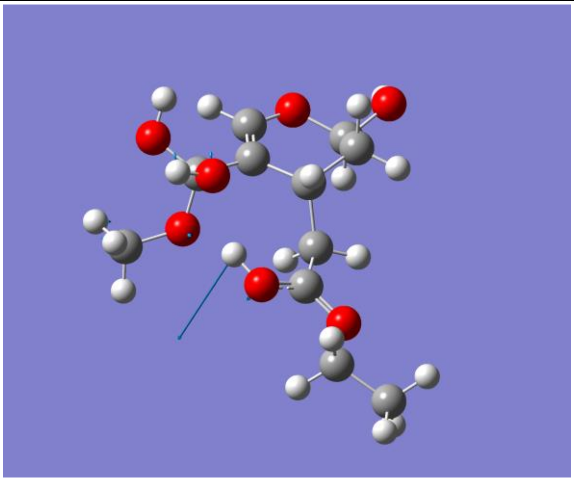	Calculation Type	TS/FREQ
	Calculation Method	RB3LYP
	Basis Set	6-31+G(d)
	Charge	1
	Spin	Singlet
	E(RB3LYP)	-956.93704
	RMS Gradient Norm	6.905e-06
	Imaginary Freq	1
	Dipole Moment	7.9339471
	Point Group	C1
**Compound: TS-3**		
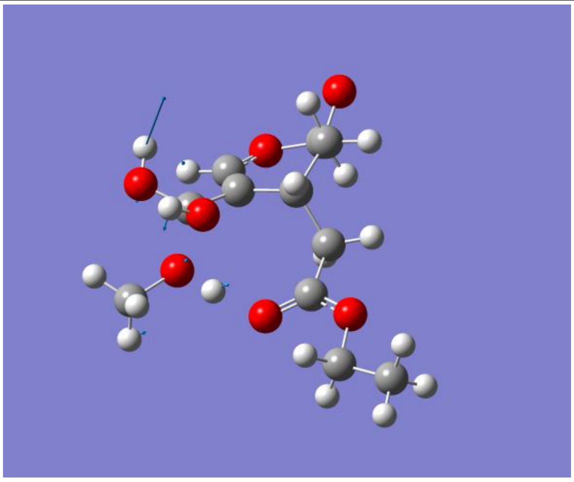	Calculation Type	TS/FREQ
	Calculation Method	RB3LYP
	Basis Set	6-31+G(d)
	Charge	1
	Spin	Singlet
	E(RB3LYP)	-956.94605
	RMS Gradient Norm	2.932e-06
	Imaginary Freq	1
	Dipole Moment	3.1694894
	Point Group	C1
**Compound: TS-4**		
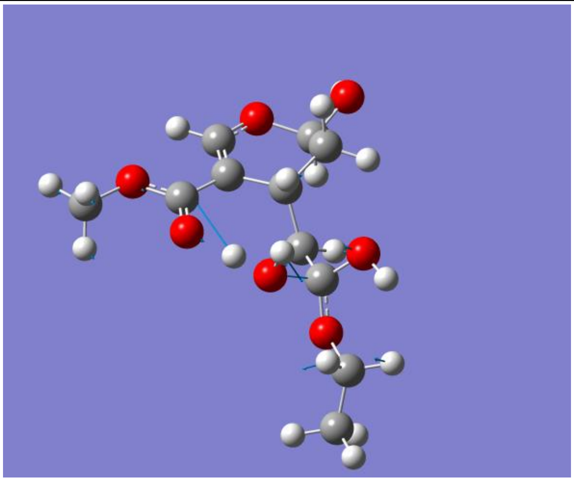	Calculation Type	TS/FREQ
	Calculation Method	RB3LYP
	Basis Set	6-31+G(d)
	Charge	1
	Spin	Singlet
	E(RB3LYP)	-956.95346
	RMS Gradient Norm	1.26e-06
	Imaginary Freq	1
	Dipole Moment	4.4081513
	Point Group	C1

Compound: TS-5		
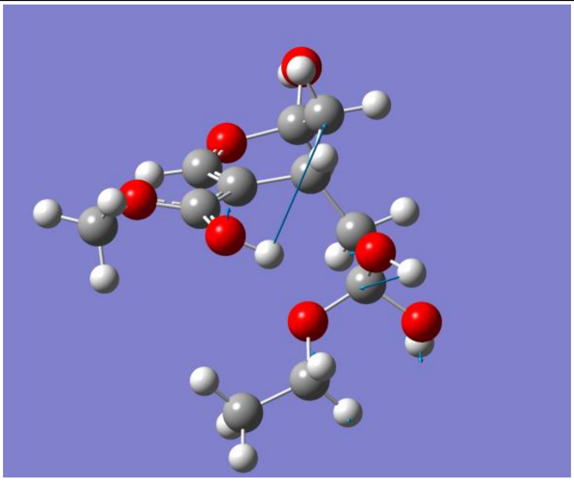	Calculation Type	TS/FREQ
	Calculation Method	RB3LYP
	Basis Set	6-31+G(d)
	Charge	1
	Spin	Singlet
	E(RB3LYP)	-956.956
	RMS Gradient Norm	5.399e-06
	Imaginary Freq	1
	Dipole Moment	2.4837113
	Point Group	C1
**Compound: TS-6**		
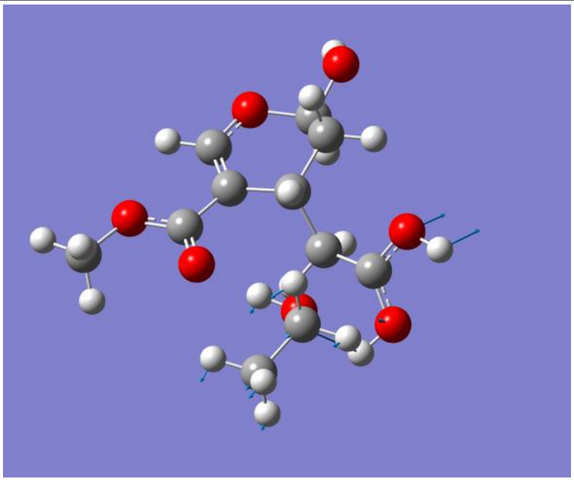	Calculation Type	FREQ
	Calculation Method	RB3LYP
	Basis Set	6-31+G(d)
	Charge	1
	Spin	Singlet
	E(RB3LYP)	-956.95409
	RMS Gradient Norm	2.18e-06
	Imaginary Freq	1
	Dipole Moment	5.9161951
	Point Group	C1
**Compound: 16**		
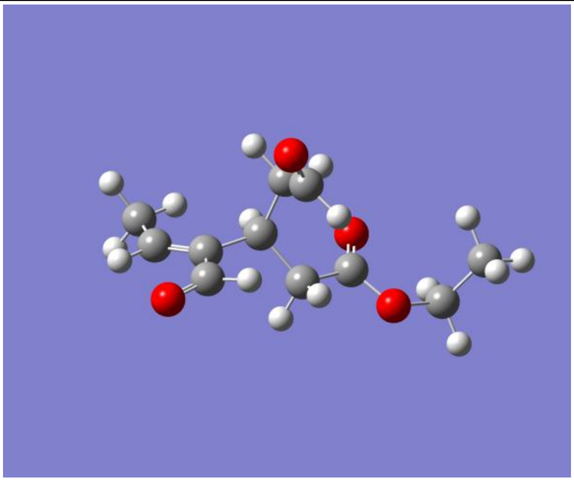	Calculation Type	FOPT
	Calculation Method	RB3LYP
	Basis Set	6-31+G(d)
	Charge	0
	Spin	Singlet
	E(RB3LYP)	-729.70246116
	RMS Gradient Norm	0.00000209
	Imaginary Freq	5.3843
	Dipole Moment	C1
	Point Group	FOPT

Compound: 16+DMSO		
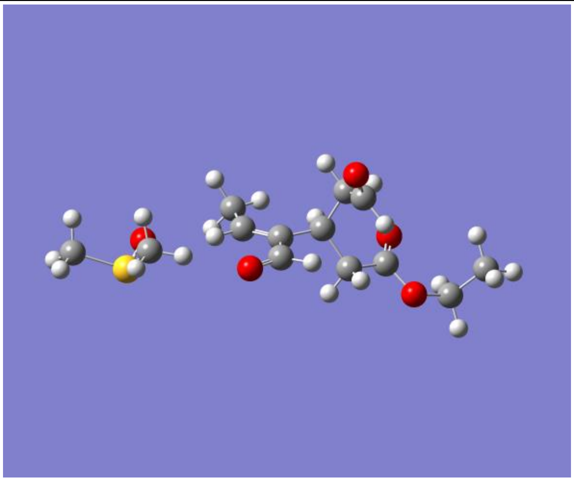	Calculation Type	FOPT
	Calculation Method	RB3LYP
	Basis Set	6-31+G(d)
	Charge	0
	Spin	Singlet
	E(RB3LYP)	-1282.91033647
	RMS Gradient Norm	0.00000235
	Imaginary Freq	2.7719
	Dipole Moment	C1
	Point Group	FOPT
**Compound: 16+2DMSO**		
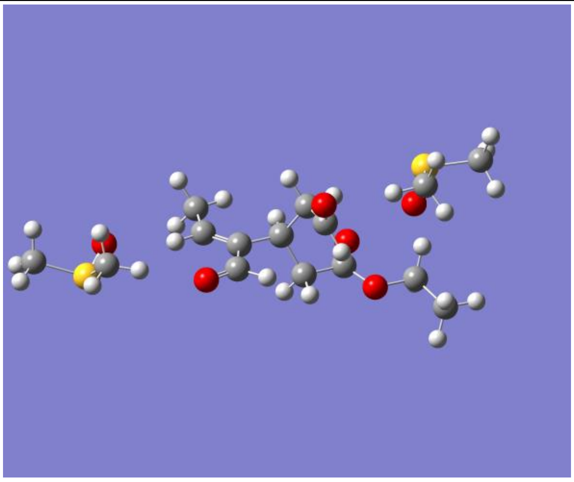	Calculation Type	FOPT
	Calculation Method	RB3LYP
	Basis Set	6-31+G(d)
	Charge	0
	Spin	Singlet
	E(RB3LYP)	-1836.11922226
	RMS Gradient Norm	0.00000081
	Imaginary Freq	3.5221
	Dipole Moment	C1
	Point Group	FOPT

References

1. Impellizzeri, J. & Lin, J. A simple high-performance liquid chromatography method for the determination of throat-burning oleocanthal with probated antiinflammatory activity in extra virgin olive oils. *J. Agric. Food Chem.* **54,** 3204–3208 (2006).
2. Adhami, H. R. *et al.* Preparative isolation of oleocanthal, tyrosol, and hydroxytyrosol from olive oil by HPCCC. *Food Chem.* **170,** 154–159 (2015).
3. Morokura, K. *et al.* Nucleophilic Substitution Reactions of Alcohols with Use of Montmorillonite Catalysts as Solid Brønsted Acids. *J. Org. Chem.* **72**, 6006–6015 (2007).
4. Jemai, H., Feki, A. E. L. & Sayadi, S. Antidiabetic and antioxidant effects of hydroxytyrosol and oleuropein from olive leaves in alloxan-diabetic rats. *J. Agric. Food Chem.* **57,** 8798–8804 (2009).
5. Samara, P. *et al.* New semi-synthetic analogs of oleuropein show improved anticancer activity in vitro and in vivo. *Eur. J. Med. Chem.* **137,** 11–29 (2017).
6. Vougogiannopoulou, K. *et al.* One-step semisynthesis of oleacein and the determination as a 5-lipoxygenase inhibitor. *J. Nat. Prod.* **77,** 441–445 (2014).
